# Advances and challenges in identifying precursors of memory CD4^+^ T cells

**DOI:** 10.3389/fimmu.2025.1540045

**Published:** 2025-05-05

**Authors:** Jianguo Liu, Wengang Song, Hua Tang

**Affiliations:** ^1^ Shandong Province University Clinical Immunology Translational Medicine Laboratory, The First Affiliated Hospital of Shandong First Medical University & Shandong Provincial Qianfoshan Hospital, Jinan, Shandong, China; ^2^ School of Clinical and Basic Medicine, Shandong First Medical University & Shandong Academy of Medical Sciences, Jinan, Shandong, China; ^3^ Institute of Infection and Immunity, Medical Science and Technology Innovation Center, Shandong First Medical University & Shandong Academy of Medical Sciences, Jinan, Shandong, China

**Keywords:** CD4 + T memory cells, memory precursor, fate decision, T memory subsets, Th1/2/17 and Tfh subsets

## Abstract

Memory T (T_M_) cells play critical roles in protective immunity and immunopathology, and their generation and maintenance have attracted a lot of interests. In recent decades, informative investigations into CD8^+^ T_M_ cell precursors have greatly enhanced our understanding of fate decision during CD8^+^ T_M_ cell differentiation. Yet, much less is known about the generation of CD4^+^ T_M_ cells and their precursors. In this review, we present advances in identifying precursors of CD4^+^ T_M_ cells under Th1, Th2 and Th17 conditions, as well as current understanding of how intrinsic factors, extrinsic factors and positioning profiles contribute to determining fate choices of CD4^+^ T cells between effector and memory. However, the path toward a general theory of CD4^+^ T_M_ cell generation has been hindered by technological limitations and diversity and plasticity of CD4^+^ T subsets at effector and memory phases. We thoroughly discuss the differences and similarities in differentiation of CD4^+^ T_M_ cells under Th1, Th2, and Th17 conditions, and explore the prospects for identifying common precursors of specific CD4^+^ T_M_ cells under various types of infections and exposures.

## 1 Introduction

**Figure 1:**
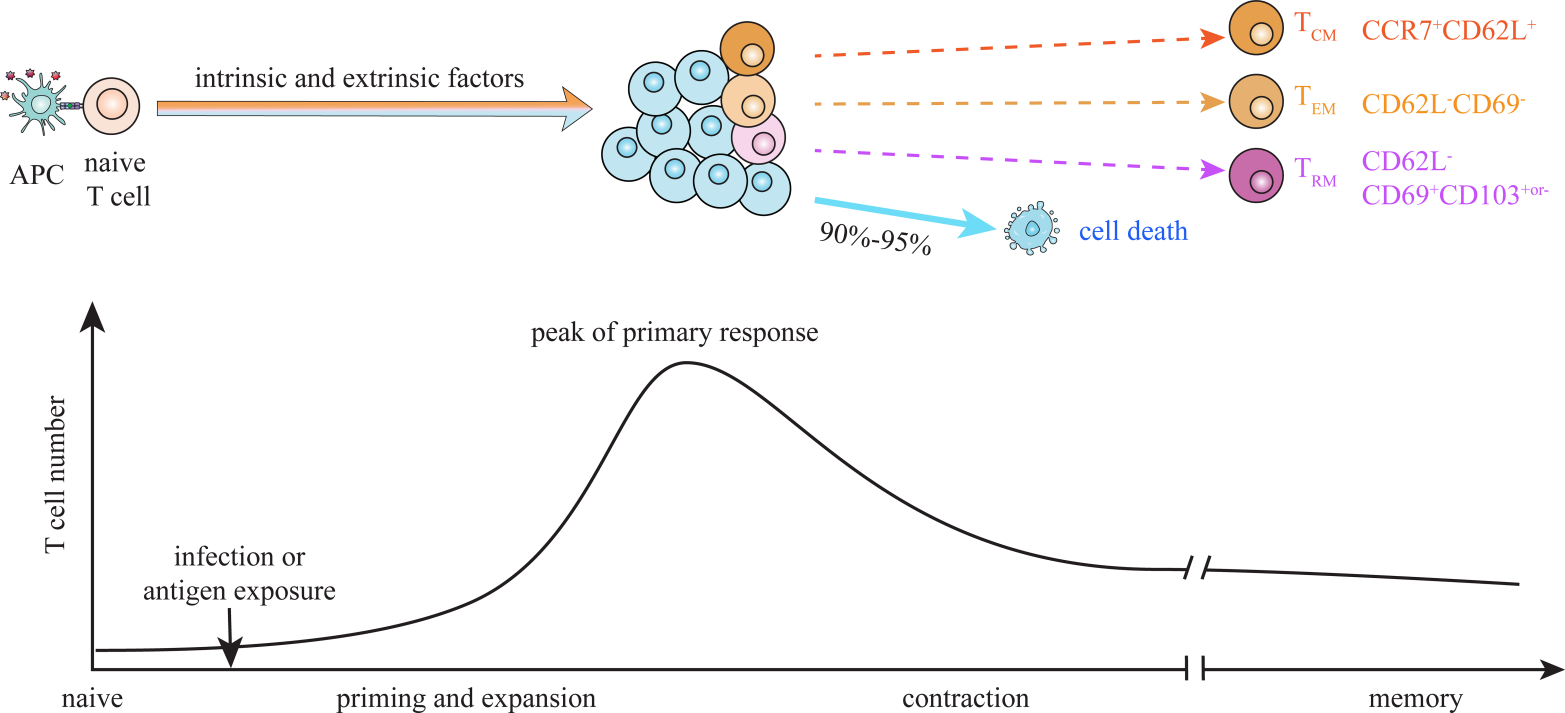
Dynamics of T cell memory formation. Upon infection or antigen exposure, naïve T cells are primed and activated by antigen-presenting cells (APCs) through pMHC-TCR interaction. Activated T cells vigorously expand and differentiate into functional effector cells during priming, and contract after antigen clearance. A small proportion of antigen-activated T cells survive contraction and become long-lived memory T (T_M_) cells. The differentiation fate of activated T cells between effector and memory is suggested to be dictated by dynamic interaction of multiple intrinsic and extrinsic factors during priming stage. Heterogeneous populations at the peak of primary response exhibit different potential to give rise to terminal effector cells and T_M_ cells. Central memory (T_CM,_ CD62L^+^CCR7^+^), effector-like memory (T_EM_, CD62L^-^CD69^-^) and tissue resident memory (T_RM_, CD62L^-^CD69^+^CD103^+or-^) cells are suggested to derive from distinct progenitors, which emerge at priming and expansion stage.

In response to pathogen infection or antigen exposure, naive T cells primed by antigen-presenting cells proliferate and differentiate into functional effector T cells. Following elimination of immunologic threat, major effector cells (about 90-95%) die during contraction phase, and only a small proportion survives and develops into long-lived memory T (T_M_) cells which are capable of self-renewal and surviving in the absence of further antigen stimulation (**Figure 1**) (1–3). CD8^+^ and CD4^+^ T_M_ cells can be typically subdivided into CD62L^+^CCR7^+^central memory T (T_CM_) cells, CD62L^-^CCR7^-^ effector-like memory T (T_EM_) cells and CD69^+^CD103^+or-^ tissue-resident memory T (T_RM_) cells based on their functions and migration patterns (4, 5). As T_M_ cells play critical roles in protective immunity and immunopathology, elucidating mechanisms underlying their generation and maintenance is essential for the design of future vaccines capable of eliciting T cell-based immunity (6).

**Figure 2:**
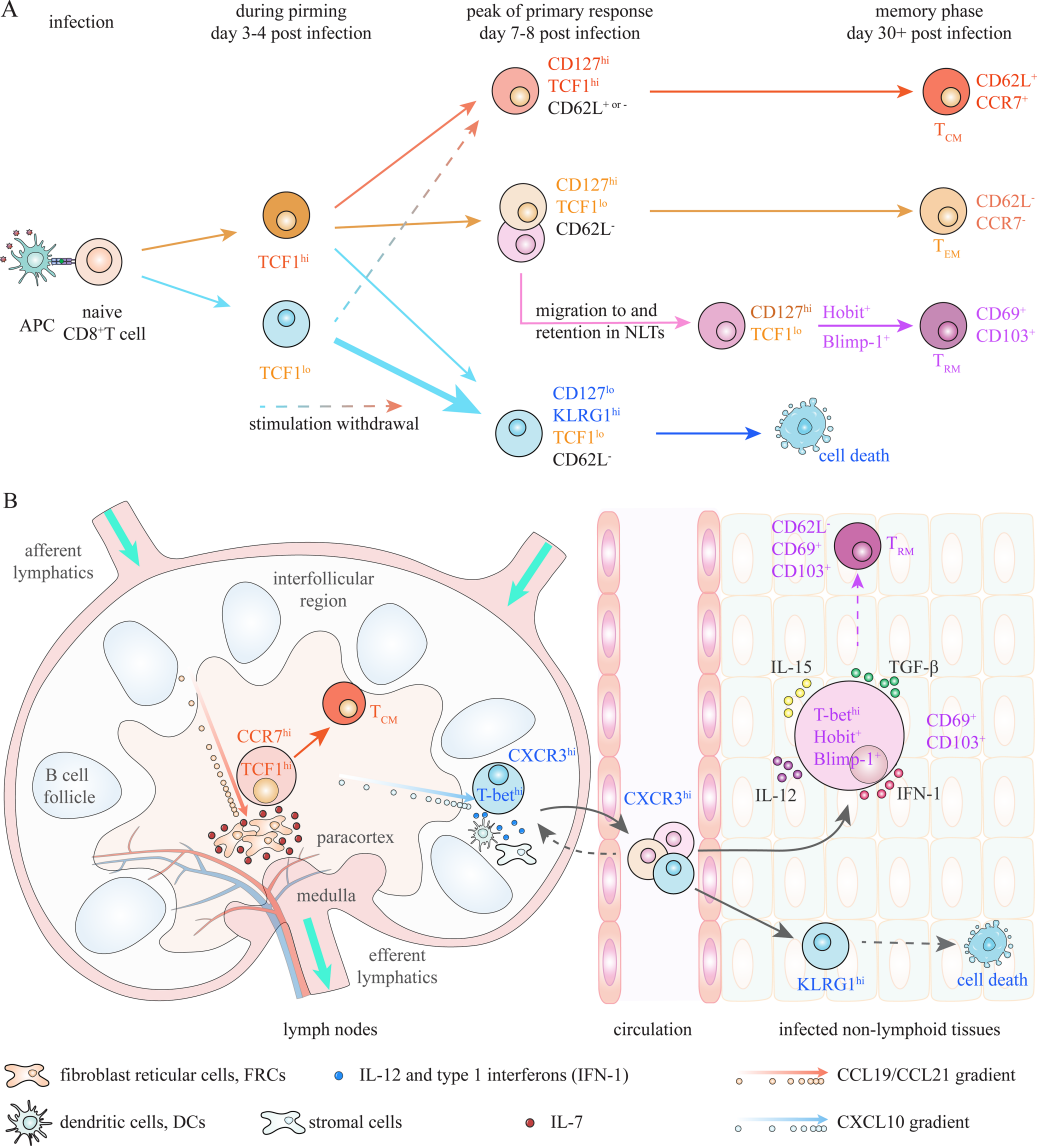
Advancing insights into precursors of CD8^+^ T_M_ cells. **(A)** At the peak of primary response, activated CD8^+^ T cells can be subdivided into CD127^hi^KLRG1^lo^ memory precursor effector cells (MPECs), which possess greater potential to generate T_M_ cells, and KLRG1^hi^CD127^lo^ short-lived effector cells (SLECs), which mostly dismiss during contraction. TCF1^hi^ cells within CD127^hi^ MPEC pool, which substantially survive contraction and generate T_CM_ cells, are identified as T_CM_ precursors. Meanwhile, circulating effector-like memory T (T_EM_) cells and tissue-resident memory T (T_RM_) cells are suggested to derive from TCF1^lo^CD127^hi^ progenitors. Moreover, the diversification of T cell fate is suggested to occur at even earlier stage. Activated TCF1^hi^ and TCF1^lo^ T cells at day 3–4 post-infection already possess distinct memory potential; however, they exhibit plasticity when microenvironment changes. Fate commitment of CD8^+^ T cells between effector and memory depends on dynamic interactions of intrinsic and extrinsic factors during whole priming stage. **(B)** Localization in proper niches to encounter appropriate extrinsic factors is also critical for effector and memory differentiation. Localization of TCF1^hi^CD127^hi^ T_CM_ precursors in the paracortex (T cell zone) of lymphoid nodes (LNs), where there is high amount of IL-7 produced by fibroblast reticular cells (FRCs), through CCR7 mediated chemoattraction towards FRC-derived CCL19/CCL21, is essential for generation of CD8^+^ T_CM_ cells. Meanwhile, CXCR3-mediated migration of CD8^+^ T cells to interfollicular region of LNs and non-lymphoid tissues (NLTs), through chemotaxis towards CXCL10, facilitates their interaction with high amount of antigen and inflammatory signals, which is essential for effector differentiation. Moreover, optimal T_RM_ generation depends on interaction with local extrinsic factors in NLTs. Expression of CD69 and/or CD103, which is regulated by several intrinsic (T-bet, Hobit and Blimp-1) and extrinsic (TGF-β) factors, is suggested to be required for retention of activated CD8^+^ T cells in NLTs.

The core theories of memory generation are primarily derived from studies on differentiation of CD8^+^ T_M_ cells (1, 7). The identification of CD127^hi^KLRG1^lo^ memory precursor effector cells (MPECs) and CD127^lo^KLRG1^hi^ short-lived effector cells (SLECs) at the peak of primary response (day 7–8 post-infection) provides a guiding framework for a deeper understanding of the fate decision between effector and memory CD8^+^ T cells (**Figure 2**) (8–11). TCF1^hi^ cells within CD127^hi^ MPEC pool, which exhibit stem-like properties and undergo less cytotoxic differentiation, substantially give rise to T_CM_ cells, while TCF1^lo^CD127^hi^ population contracts and becomes T_EM_ cells following infection elimination (3, 12–14). Preferential localization of TCF1^hi^ T_CM_ precursors in paracortex (T cell zone) of secondary lymphoid organs (SLOs), through CCR7-mediated chemotaxis toward CCL19/21, facilitates their encounter with IL-7, thus enhancing their transition into CD8^+^ T_CM_ cells (**Figure 2**) (15–20). Meanwhile, T-bet-induced CXCR3 drives the migration of activated CD8^+^ T cells to peripheral region of SLOs and inflamed non-lymphoid tissues (NLTs), facilitating their interaction with inflammatory signals and thereby promoting their differentiation into CD127^lo^KLRG1^hi^ terminal effector cells (9, 19, 21). CD69^+^CD103^+^ CD8^+^ T_RM_ cells are suggested to derive from a MPEC-like (CD127^hi^) population residing in NLTs, and Hobit and Blimp-1 potentially identify T_RM_ precursors in different NLTs (22–25). Moreover, early-activated TCF1^hi^ and TCF1^lo^ cells (day 2–4 post-infection), which already possess distinct memory potential, exhibit reversible plasticity between effector and memory fates in response to inflammatory stimulation or the withdrawal of such stimulation (12, 14, 26, 27). The differentiation fate of antigen-activated CD8^+^ T cells between effector and memory is collaboratively determined by intrinsic factors, extrinsic factors and positioning profiles that facilitate intrinsic-extrinsic interactions during the whole priming phase under acute infection (1, 3, 28–31).

Though there is no unifying framework, the current understanding of CD8^+^ T_M_ cell generation provides a valuable guidance for investigations into differentiation of CD4^+^ T_M_ cells. However, much less is known about generation of CD4^+^ T_M_ cells and their precursors to date (5, 32). Unlike CD8^+^ T cells, antigen-activated CD4^+^ T cells can differentiate into Th1, Th2, and Th17 effector cells, which are typically identified by secretion of signature cytokines, under different types of infections and exposures (33). Th1 cells, characterized by IFNγ secretion, are induced by the master transcription factor T-bet in response to intracellular viral and bacterial infections. IL-4-secreting Th2 cells are promoted by transcription factor Gata3 upon exposure to extracellular parasites/helminths and allergens. The differentiation of IL-17-secreting Th17 cells depends on expression of transcription factor RORγt in response to fungal and extracellular bacterial infections. Additionally, Th1/2/17 responses are all associated with Bcl6-dependent parallel differentiation of CXCR5^+^PD-1^+^ follicular helper T (Tfh) cells, which play pivotal roles in promoting B cell responses within germinal center (GC) (34). Th1/2/17 and Tfh cells both could survive contraction and become T_M_ cells after immunological threat is eliminated (5, 35–39). The heterogeneity of activated CD4^+^ T cells adds complexity to studying the generation of CD4^+^ T_M_ cells.

Drawing on insights from the theory of CD8^+^ T_M_ cell generation, this paper presents current advances in identifying precursors of Th1, Th2, Th17, and Tfh memory cells, as well as elucidating the mechanisms underlying fate decision of CD4^+^ T_M_ cells. We also discuss the challenges and prospects for identifying common precursor of specific CD4^+^ T_M_ cells under various types of infections and exposures.

## 2 Advancing insights into CD4^+^ T_M_ cell precursors during Th1 responses

### 2.1 Diverse effector and memory CD4^+^ T cell subsets

**Figure 3:**
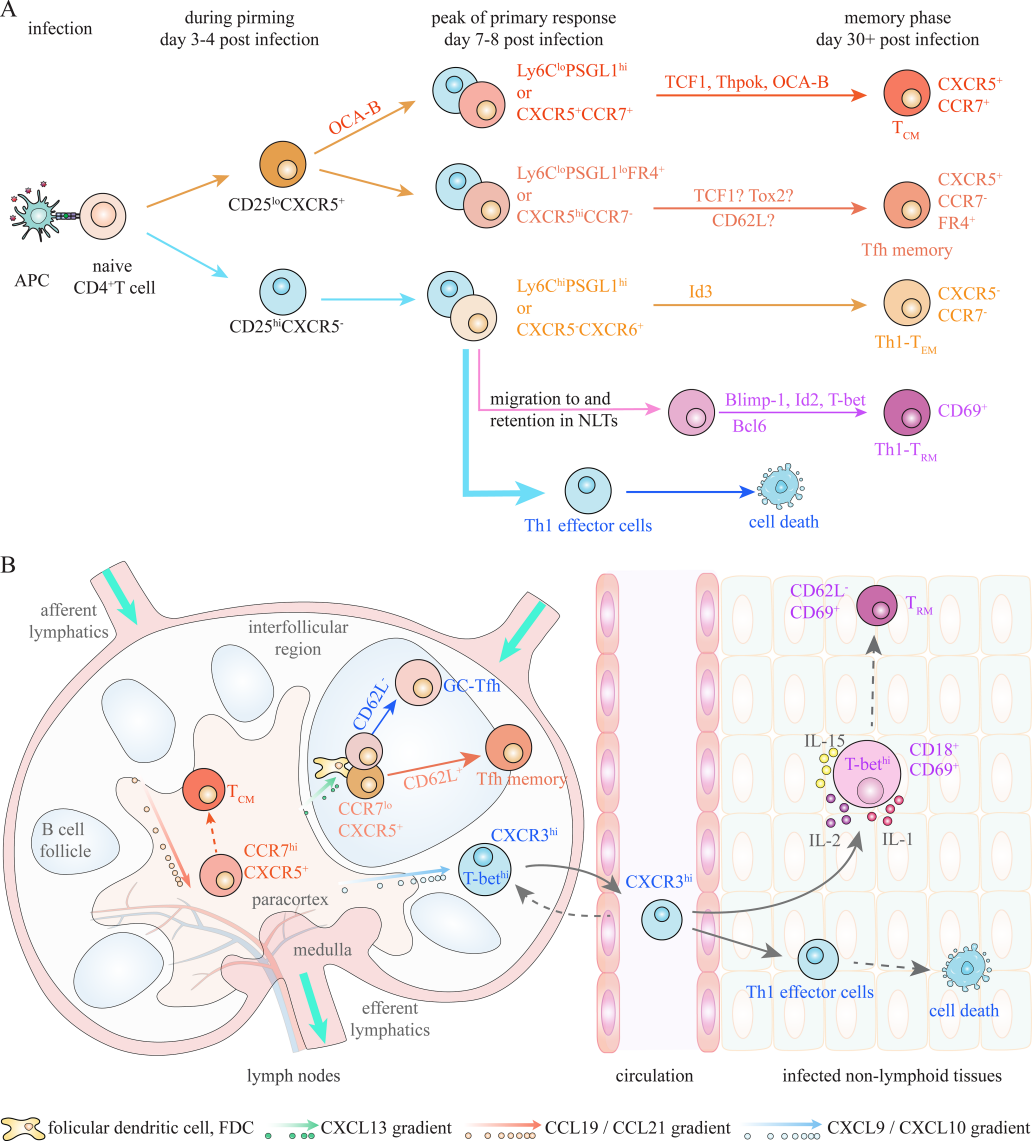
Potential memory precursors during Th1/Tfh responses under infection. **(A)** Activated CD4^+^ T cells at the peak of primary response can be subdivided into several populations, which enrich precursors of T_CM_, Th1-T_EM_, Th1-T_RM_ and Tfh memory cells respectively, based on expression of Ly6C/PSGL1/FR4 and/or CXCR5/CCR7/CXCR6. Multiple factors have been revealed to regulate generation of CD4^+^ T_M_ cells during Th1/Tfh responses under bacterial and viral infections, and thus can act as putative marker for memory precursors. Moreover, IL-2/CD25 signaling plays critical role in determining fate choices between Th1 effector and Tfh/T_CM_ cells at early stage of priming. **(B)** Spatial localization of CD4^+^ T cells also contributes to effector and memory differentiation under infections. CCR7 retains activated CD4^+^ T cells in paracortex (T cell zone), despite co-expression of CXCR5, which is essential for generation of T_CM_ cells. Migration to interfollicular region and entry into peripheral non-lymphoid tissues (NLTs), which is mediated by CXCR3-CXCL9/10 chemotaxis, promotes optimal Th1 effector differentiation. NLTs residency of activated T cells, which is partly regulated by CD69 and T-bet-induced CD18, is required for Th1-T_RM_ generation. CXCR5-mediated positioning in germinal center **(GC)** is required for GC-Tfh and Tfh memory cell differentiation. A CD62L^+^PD-1^lo^ subpopulation of Tfh-like (CXCR5^+^BCL6^+^) cells, which exhibit less preference to interact with B cells, is suggested to efficiently generate Tfh memory cells.

Consistent with CD8^+^ T_M_ subsets, CD4^+^ T_M_ cells can be traditionally categorized into CD62L^+^CCR7^+^ T_CM_ cells, CD62L^-^CCR7^-^ T_EM_ cells, and CD69^+^ T_RM_ cells under bacterial and viral infections (**Figure 3**) (36, 40, 41). T_CM_ cells, which circulate through lymph nodes, can efficiently generate secondary Th1 effectors upon rechallenge, while T_EM_ cells, which migrate between NLTs, are able to rapidly produce IFNγ following rechallenge (36, 37, 41, 42). T_RM_ cells, which mainly reside in NLTs, mediate rapid local recall responses (43). Additionally, CCR7^-^CXCR5^+^ Tfh memory cells, which are essential for secondary B cell responses, have been identified as a distinct memory population, although they exhibit phenotypic similarities with CCR7^+^CXCR5^+^ T_CM_ cells, including the expression of TCF1, CD127 and CXCR5 (37, 41, 44–46). The diverse T_M_ subsets are suggested to derive from relevant effector cell populations at peak of primary responses (35, 39). Currently, two commonly used approaches, based on expression of CXCR6/CXCR5/CCR7 and Ly6C/PSGL1/FR4, are employed to identify effector and memory populations by flow cytometry.

The first approach subdivides activated CD4^+^ T cells into CXCR6^+^CXCR5^-^ Th1-like cells and CXCR6^-^CXCR5^+^ Tfh-like cells at the peak of immune response during viral infection (37, 39, 41). CXCR5^-^CXCR6^+^T-bet^+^ IFNγ-secreting Th1 effector cells can partially survive contraction and give rise to CXCR5^-^CCR7^-^ Th1-T_EM_ cells (36, 38, 39, 41). CCR7^+^ cells within CXCR5^+^ Tfh-like population at effector phase, which exhibit a less differentiated state, primarily develop into T_CM_ cells identified by the CCR7^+^CXCR5^+^ phenotype (37, 41, 42). CXCR5^+^CCR7^-^ Tfh cells during the priming phase can give rise to CXCR5^hi^PD-1^hi^ GC-Tfh cells and CXCR5^+^CCR7^-^ Tfh memory cells (39, 46, 47). However, CXCR5 expression on Tfh cells has been shown to decrease or even disappear during memory phase; therefore, CXCR5^-/lo^ T_M_ cells might actually contain Tfh memory cells (37, 42, 45). In addition, CXCR5^-/lo^ T_M_ cells also contain a CCR7^+^ T_CM_-like population (36, 41, 42). This suggests a certain degree of unreliability of this approach in identifying above CD4^+^ T cell subsets during effector and memory phases.

Another approach partitions activated CD4^+^ T cells into Ly6C^hi^PSGL1^hi^, Ly6C^lo^PSGL1^hi^, and Ly6C^lo^PSGL1^lo^ populations at effector phase, and these populations are also observed at memory stage (44, 45, 48, 49). Ly6C^hi^PSGL1^hi^ population primarily consist of differentiated Th1 effector cells, which can give rise to Ly6C^hi^ Th1-T_EM_ cells (44, 45, 48, 49). Ly6C^lo^PSGL1^lo^ population, with exclusively high expression of FR4, contains Tfh cells at both effector and memory phases (37, 45, 48). Single-cell transcriptomic experiments suggest that FR4 is a reliable marker for distinguishing effector and memory Tfh cells from CCR7^+^ T_CM_ cells and their precursors, while CXCR5 is expressed on both Tfh and non-Tfh cells (37, 45). Ly6C^lo^PSGL1^hi^ effector population, which exhibits greater memory potential, is a heterogenous population comprising CXCR5^-^ Th1-like cells and CXCR5^+^ Tfh-like cells (44, 45). This population may contain T_CM_ precursors, as CD62L^+^CCR7^+^ T_CM_ cells are preferentially enriched in the Ly6C^lo^PSGL1^hi^ T_M_ cells at memory phase (37, 44, 45, 48).

Based on the above information, activated CD4^+^ T cells at the peak of priming consist of at least three populations: CXCR5^-^CXCR6^hi^Ly6C^hi^ Th1 effector cells, CCR7^+^CXCR5^+^ cells enriched for T_CM_ precursors, and CCR7^-^CXCR5^+^FR4^+^ Tfh cells (37, 39, 41, 45). It is suggested that a proportion of cells in each population survive contraction and become CD4^+^ T_M_ cells following infection elimination (35, 39, 41, 42). Additional heterogeneity clearly exists within these populations; however, mechanisms driving their transition into CD4^+^ T_M_ cells remain under investigation.

### 2.2 Intrinsic factors that determine fate decision between effector and T_M_ cells

Fate mapping studies have demonstrated that single naïve CD4^+^ T cells can give rise to effector and memory Th1 and Tfh cell populations during infection (38, 50). Similar to CD8^+^ T cells, differentiation fate of CD4^+^ T_M_ cells is suggested to be determined during the priming phase of infection (32, 39, 41). A microarray study indicates that the generation of CD4^+^ T_M_ cells relies on a combination of programs that inhibit proliferation and apoptosis while promoting DNA damage repair and lipid metabolism during Th1 response in malaria infection (51). Multiple factors have been revealed to be essential for CD4^+^ T_M_ cell differentiation.

It is well-established that CD127 and TCF1 play decisive roles in CD8^+^ T_M_ cell differentiation (3, 8, 9, 12–14). However, CD127 expression is unable to distinguish CD4^+^ T_M_ cell precursors from terminal Th1 effector cells, although it is required for maintenance and homeostasis of CD4^+^ T_M_ cells (20, 37, 41, 45, 48, 52). TCF1 has been shown to be involved in maintaining stemness and promoting formation of T_CM_ cells; however, it also promotes Tfh polarization and inhibits Th1 effector differentiation under viral infections (39, 52–57). Another T cell intrinsic factor, Thpok, which is essential for the differentiation of CCR7^+^ T_CM_ precursors, also plays critical role in Tfh cell differentiation under lymphocytic choriomeningitis virus (LCMV) infection (41, 58). Therefore, neither TCF-1 nor Thpok can faithfully identify CD4^+^ T_CM_ precursors. In addition, Bcl6, which is indispensable for Tfh differentiation, contributes to the generation of CCR7^+^ T_CM_ cells primarily through repressing Blimp-1 expression (42, 59, 60). Id3 also contributes to generation of CXCR5^+^CCR7^+^ T_CM_ cells and CXCR5^+^CCR7^-^ Tfh-like memory cells under LCMV infection (36). It is challenging to identify reliable precursors of T_CM_ cells due to the shared programs underlying differentiation of T_CM_ and Tfh cells. Recently, OCA-B (*Pou2af1*), which increases Th1-like cells and reduces Tfh-like cells during priming, has been shown to be necessary and sufficient to drive the generation of CD44^+^CD62L^+^ T_CM_ cells under LCMV infection, and thus can prospectively identify T_CM_ precursors (61).

Meanwhile, Th1-T_EM_ cells have been shown to derive from *in vitro* activated IFNγ-secreting Th1-like effector cells (62, 63). Expression of CD30 (*Tnfrsf8*) and inhibition of ACC1 are demonstrated to enhance generation of Th1 memory cells, although these studies did not investigate Tfh cells and diverse T_M_ lineages (64, 65). A recent study reveals that expression of Id3 identifies a memory precursor-like population within CXCR5^-^ Th1-like cells, which can survive contraction phase and become CXCR5^-^CCR7^-^ Th1-like T_EM_ cells (36).

Furthermore, TCR-dependent CD25 (IL-2Rα) expression is suggested to predict the differentiation fate of activated CD4^+^ T cells as early as day 3 post-infection (**Figure 3A**) (42, 57, 66). Indeed, IL-2/IL-2R signaling have been demonstrated to play a crucial role in determining fate choices between effector and memory CD8^+^ T cells (67). Following the initial induction of CD25 after TCR activation, its rapid downregulation on activated CD8^+^ T cells during priming and diminished IL-2 signaling is essential for generation of CD8^+^ T_M_ cells, whereas prolonged IL-2/CD25 signaling promotes terminal effector differentiation (67). Similarly, early-activated CD25^hi^ CD4^+^ T cells (day 3 post-infection), which lack CXCR5 expression, almost exclusively develop into terminal Th1 effector cells, whereas CD25^lo^ cells, which generally express CXCR5, give rise to Tfh and T_CM_ cells (42, 57, 66, 68). Moreover, early-activated CD25^lo^ cells are predominantly enriched for OCA-B^hi^ cells, although a proportion of CD25^hi^ cells also express lower level of OCA-B (61). It is indicated that high expression of OCA-B at the early stage of priming contributes to the commitment of CD25^lo^ cells to a T_CM_ cell fate. However, whether early-activated CD25^hi^ and CD25^lo^ CD4^+^ T cells exhibit plasticity in response to changes in the microenvironment remains to be further investigated. Notably, it has been demonstrated that IL2/CD25 signaling promotes survival of activated T cells and thus formation of T_CM_ and T_EM_ cells, through upregulating re-expression of CD127 during contraction phase (69–71). Nevertheless, IL-2/CD25 signaling at early stage of priming contributes to determining the differentiation fates between Th1 effector cells and Tfh/T_CM_ cells during Th1 response (57).

### 2.3 Positioning profiles that dictate differentiation fate between effector and T_CM_ cells

As well as intrinsic factors, spatially distributed antigens and cytokines within microenvironment also play essential roles in CD4^+^ T effector and memory differentiation (**Figure 3B**) (32, 33). Inflammatory cytokines within inflamed NLTs, such as IL-12 and IFNγ, are critical for the differentiation of IFNγ-secreting Th1 effector cells, partly through promoting T-bet expression (72–76). Meanwhile, high level of IL-7 in SLOs produced by fibroblast reticular cells (FRCs) promotes generation of CD4^+^ T_CM_ cells, although its role in this process is not as prominent as in the formation of CD8^+^ T_CM_ cells (20). T-bet-induced CXCR3 expression on activated CD4^+^ T cells mediates their migration rapidly out of lymph organs and into inflamed peripheral NLTs, thereby promoting optimal Th1 effector differentiation during influenza virus (IAV) infection (77, 78). Meanwhile, upregulation of CD62L in TCF1^hi^ cells leads to their enrichment in SLOs rather than accumulation in lungs, and thus contributes to T_CM_ cell formation during Th1 response in response to IAV infection (52).

Moreover, precise localization of activated CD4^+^ T cells within SLOs also contributes to determining their fate choices between effector and memory (**Figure 3B**). Peripheral region within secondary lymphoid organs (SLOs) provides abundant antigen and inflammatory cytokines (IL-12, and IFNγ), while IL-7-producing fibroblast reticular cells (FRCs) are restricted in the center (T cell zone) of SLOs (7, 79, 80). CXCR3 directs the migration of activated CD4^+^ T cells to interfollicular region of LNs, which is strongly correlated with increased Th1 effector differentiation under viral infection (81, 82). Retention of activated T cells in the T cell zone, regulated by CCR7-mediated chemotaxis towards FRC-produced CCL19/CCL21, facilitates their encounter with high amount of IL-7 and protects them from excessive inflammatory stimulation, thus promoting CD4^+^ T_CM_ generation during Th1 responses (15, 20, 83, 84). In line with this, CCR7^+^CXCR5^+^ T cells, which enrich precursors of CD4^+^ T_CM_ cells, predominantly localize in T cell area of SLOs (37, 41, 42). Additionally, Ly6C^lo^PSGL1^hi^ cells with greater memory potential also preferentially localize in T cell zone, while Ly6C^hi^ cells, which mainly give rise to terminal Th1 effectors, migrate to peripheral sites of spleen under LCMV infection (48).

Similar to CD8^+^ T cells, retention in T cell area of SLOs is required for CD4^+^ T_CM_ formation, whereas migration to periphery of SLOs and entry into NLTs promote Th1 effector differentiation. The balance between CCR7 and CXCR3 expression contributes to dictating differentiation fate between CD4^+^ T_CM_ and Th1 effector cells. Yet, spatial requirement of Th1-T_EM_ cell generation remains unclear.

### 2.4 Identifying precursors of Tfh memory cells during Th1 responses

There are also several factors that have recently been shown to be involved in generation of Tfh memory cells during Th1 responses. In addition to its role in Tfh lineage polarization, TCF1 has been shown to be essential for the generation and maintenance of Tfh memory cells (52–56). Tox2 is also required for GC-Tfh differentiation and Tfh memory cell generation under IAV infection (85). Moreover, PD-1^+^CXCR5^+^ Tfh-like cells with sustained expression of Tigit preferentially differentiate into GC-Tfh cells, although Tigit is not functionally critical for differentiation and function of GC-Tfh cells (84). Meanwhile, Tigit-negative Tfh-like cells upregulate CD127 expression by day 14 post-infection (after GC formation) and give rise to CXCR5^+^ T_M_ cells with or without CCR7 expression (84). Recently, a CD62L^+^PD-1^lo^ subpopulation within CXCR5^+^BCL6^+^ Tfh cells, which highly expresses KLF2 and CD127, exhibits memory precursor-like transcriptional profiles and readily generates PD-1^hi^ Tfh effector cells upon recall (86). In addition, the CD62L^+^PD-1^lo^ population exhibits a reduced preference for B cell interaction (86). The observation is consistent with the suggestion that avoiding excessive stimulation from B cells is essential for Tfh memory cell generation (46, 84, 85). Although functional requirements of Tigit and CD62L in generation of Tfh memory cells is unclear, they can putatively act as phenotypic markers for distinguishing between progenitors of GC-Tfh and Tfh memory cells.

Spatial requirement for development of Tfh memory cells appears to be different from T_CM_ and Th1 effector cells. Expression of CXCR5, which facilitates positioning of pre-Tfh cells at T:B border and entry into follicle to appropriately interact with B cells, is essential for further GC-Tfh and Tfh memory cell differentiation (84, 87–91). Upregulation of PD-1 also promotes accumulation of Tfh-like cells in the GC territory, partly through inhibiting expression of CXCR3 which can otherwise distract Tfh-like cells from GC localization (91, 92). Meanwhile, CCR7 expression retains activated CD4^+^ T cells in T cell zone and thus inhibits their differentiation towards Tfh fate, despite co-expression of CXCR5 (84, 89, 90). Expression of CXCR5, as well as downregulation of CCR7 and CXCR3, drives migration of activated CD4^+^ T cells toward B cell follicles and GCs, thus facilitating differentiation of GC-Tfh and Tfh memory cells.

### 2.5 Identifying precursors of Th1-T_RM_ cells in NLTs

Th1-T_RM_ cells, which exhibit Th1 effector and memory profiles, are suggested to derive from Th1 effector cells residing in NLTs under bacterial and viral infections (**Figure 3A**) (93, 94). IL-2/CD25 signaling has also been demonstrated to enhance Th1-T_RM_ generation in the lung through promoting optimal effector differentiation during IAV and LCMV infection (95, 96). Th1-associated T-bet, Blimp-1, and Id2 are demonstrated to be required for Th1-T_RM_ generation in liver and small intestine during *Salmonella* and LCMV infections (93, 94). Meanwhile, Hobit and Blimp-1, which plays crucial role in CD8^+^ T_RM_ cell generation, have been shown to be dispensable for CD4^+^ T_RM_ cell formation in the colon during experimental colitis, although their deficiency impairs the expression of pro-inflammatory cytokines in CD4^+^ T_RM_ cells (24, 25, 97). Differential requirements for Blimp-1 indicate that the mechanisms underlying Th1-T_RM_ formation may vary across different tissues and types of infections. On the other hand, IL-15 receptor-mediated direct IL-15 signaling within the first week promotes the generation of viral-specific Th1-T_RM_ cells through enhancing their survival and persistence in the lung during IAV infection (96). Tfh-associated Bcl6 contributes to Th1-T_RM_ generation in the small intestine under LCMV infection, probably through enhancing memory attributes (94). It is suggested that the factors contributing to effector differentiation and survival are both required for Th1-T_RM_ cell generation. However, none of these factors exhibit the ability to reliably distinguish T_RM_ precursors from terminal Th1 effector cells within NLTs.

Similar to CD8^+^ T_RM_ cells, the transition of activated CD4^+^ T cells into Th1-T_RM_ cells also depends on their interaction with local inflammatory (IL-2 and IL-1) and survival (IL-15) cytokines in microenvironment of livers and lungs during bacterial and viral infections (95, 96, 98). The factors that promote entry and retention of activated CD4^+^ T cells in NLTs are essential for the generation of T_RM_ cells. IL-2/CD25 signaling contributes to Th1-T_RM_ generation through promoting residency of activated CD4^+^ T cells in the lung during IAV and LCMV infection (95, 96). Blimp-1 has also been shown to promote the accumulation of potential early T_RM_ precursors in small intestine, thus enhancing Th1-T_RM_ generation during LCMV infection (94). T-bet-induced CXCR3, which is highly expressed on Th1 effector and T_RM_ cells, promotes entry of activated CD4^+^ T cells into liver and lung during *Salmonella* and IAV infections; however, its role in T_RM_ cell generation is unclear (77, 98). Unlike CD8^+^ T_RM_ cells, CD103 appears not to be uniformly required for the retention of activated CD4^+^ T cells in various NLTs, as it is frequently absent from Th1-T_RM_ cells (40, 43, 94, 98). CD69, which is highly expressed on Th1-T_RM_ cells in multiple NLTs, are currently regarded as a phenotypic marker for T_RM_ cells and their precursors (**Figure 3B**), although its role in NLTs residency of Th1 effector and T_RM_ cells is ambiguous (40, 95, 96, 98). Additionally, CD18, induced by highly expressed T-bet, is suggested to be essential for positioning of activated CD4^+^ T cells into liver niches and thus T_RM_ generation under *Salmonella* infection (**Figure 3B**) (93). Yet, whether CD69 and CD18 are capable of identifying precursors of Th1-T_RM_ cells within different NLTs under various types of infections remains to be further clarified. Variations in the programs governing the generation of Th1-T_RM_ cells across different types of infections and tissues increase the complexity of identifying their reliable precursors.

## 3 Potential precursors of Th2 memory cells

In comparison to Th1 cells, memory generation of Th2 cells has been much less studied. Th2 memory cells, including T_CM_ cells in lymph nodes, T_EM_ cells, T_RM_ cells in lungs, and Tfh-like memory cells all can be generated after antigen clearance under helminth/parasite infection (63, 87, 99, 100). However, Th2 and Tfh-like cells in LNs exhibit great plasticity under parasite infection, and a proportion of activated CD4^+^ T cells appear to co-express Tfh marker Bcl6/CXCR5 and Th2 marker Gata3/IL-4 (101, 102). Meanwhile, entry of activated CD4^+^T cells into lungs, which promotes terminal IL-4^+^IL-13^+^ Th2 effector cell differentiation, depends on downregulation of the hallmark Tfh transcription factor Bcl6, indicating a distinction between them (99, 101). The lack of clear delineation between Th2 and Tfh cells complicates the investigation into their memory generation under Th2 conditions.

Without considering the existence of Tfh cells, antigen-activated CD44^hi^CD62L^+^ CD4^+^T cells, which preferentially accumulate in LNs rather than lungs, are suggested to enrich T_CM_ precursors (103, 104). CD44^hi^CD62L^+^ cells, with high co-expression of CCR7, maintain a less differentiated state as indicated by lower expression of Gata3 and IL-4 (103–105). It is consistent with the observation that CCR7-mediated retention of activated CD4^+^ T cells in T cell zone inhibits Th2 effector differentiation (87, 106). Meanwhile, CXCR5-mediated migration towards peripheral region of LNs, in a CXCL13-dependent manner, is required for differentiation of both IL-4-producing Th2-like cells and Tfh cells (87, 106). It is indicated that positioning into periphery and center of SLOs also contributes to differentiation of Th2 effector cells and T_CM_ cells, respectively, during Th2 responses. On the other hand, retention of Th2 effector cells in NLTs facilitates their encounter with local extrinsic signals and thus promotes Th2-T_RM_ cell generation. Local IL-7 signaling is essential for maintenance of Th2-T_RM_ cells in lungs (105). IL-2/CD25 signaling has been shown to promote Th2-T_RM_ generation through enhancing optimal effector differentiation and residency of Th2 cells in lungs (99). CD69, which is highly expressed on Th2-T_RM_ cells, might be required for their generation through promoting lung residency of Th2 effector cells (99). Although the underlying mechanisms are not well-defined, these investigations indicate similarities in spatial requirements for memory generation between Th2 cells and Th1/CD8^+^ T cells. In addition, ablation of ACC1, which is required for Th1 memory cell formation, also enhances generation of Th2 memory cells via regulating fatty acid oxidation under helminth infection (65). It suggests that Th2 memory cell generation depends on some mechanisms shared with Th1 cells.

Despite the similarities mentioned above, it should not be simply presumed that factors involved in formation of CD8^+^ T_M_ and Th1 memory cells play the same role in Th2 memory cell generation. CD127, the key marker for identifying CD8^+^ T_M_ cell precursors, is barely expressed on Th2-like effector cells in LNs during priming and thus cannot mark Th2 memory precursors, although it has been shown to be essential for homeostasis of Th2 memory cells (104, 105). Ly6C and PSGL1, which are used to identify memory precursors from Th1 effector cells, are barely expressed on ovalbumin-specific Th2-like effector cells at the peak of Th2 responses (104). CXCR5, a key marker to distinguish between Th1 effector cells and Tfh/T_CM_ cells, is expressed on both Tfh and Th2 cells in lymph nodes during priming (87, 106). RNA-seq data also demonstrate that TCF1, Thpok and Id3, which are essential for T_CM_ and Th1-T_EM_ generation under viral infection, are comparably expressed on CD62L^-^ Th2 cells and T_CM_ precursor-enriched CD62L^+^ Th2 cells (104). Overall, independent investigations into generation of Th2 memory cells are definitely required.

## 4 Potential precursors of Th17 memory cells

Few investigations have been performed into memory generation during Th17 responses, while the heterogeneity of T_M_ cells and the existence of Tfh memory cells have remained largely unexamined. Th17 memory cells are shown to derive from IL-17^+^ or RORγT^+^ Th17-like effectors; however, how effector cells contribute to the ultimate Th17 memory cell pool remains ill-defined (107, 108). IL-7 and IL-15 have been shown to promote the maintenance of Th17 memory cells at inflammatory site and draining lymphoid tissues (109). IL-23/IL-23R signaling directly drives effector to memory conversion of Th17 cells via upregulation of CD127 and IL-15 receptor during contraction phase, while IL-2 prominently impairs IL-23-induced Th17 memory cell generation (110). It suggests that surviving signals are also required for Th17 memory generation. CD30, which promotes Th1 memory generation, plays critical role in generation of Th17 memory cells, and thus can serve as a prospective marker for Th17 memory precursors (64). Moreover, retention of Th17 cells in the lung and skin to interact with IL-1α and local IL-23 is required for CD69^+^ Th17-T_RM_ generation, although factors that mediate their residency remain unclear (111, 112). Though the information are fragmented, the differentiation of Th17 memory cells exhibits both similarities and differences with CD8^+^ T_M_ cells and Th1/2 memory cells.

## 5 Challenges to develop a general model of CD4^+^ T_M_ cell generation

Despite the above advances in characterizing precursors and elucidating fate decision mechanisms of CD4^+^ T_M_ cells, current knowledge remains insufficient. A deeper understanding of generation of CD4^+^ T_M_ cells has been hindered by several technological and biological challenges. The first challenge is to trace antigen-specific CD4^+^ T cells *in vivo*. TCR-transgenic T cell adoptive transfer system and peptide:MHC tetramer technology, which are vital tools for studying antigen-specific CD8^+^ T cells, both exhibit limitations in studying CD4^+^ T cells (5, 32). Moreover, the number and expansion capability of antigen-specific naïve CD4^+^ T cells is much lower than CD8^+^ T cells, and CD4^+^ T_M_ cells are suggested to be less stable over time (32, 42, 113, 114). Low number of antigen-specific CD4^+^ T_M_ cells makes them harder to be detected *in vivo*.

In addition to technical limitations, the functional and phenotypic heterogeneity of CD4^+^ T cells at effector and memory phase poses significant challenges to establishing a unifying framework for CD4^+^ T_M_ cell generation. As mentioned above, Th1/2/17 and Tfh populations both can give rise to T_M_ cells after immunological threat is eliminated (5, 35–39). Plenty of T cell intrinsic and extrinsic factors, which contribute to generation and maintenance of CD4^+^ T_M_ cells, are also involved in polarization of naïve T cells towards Th1/2/17 and Tfh lineages (5, 33, 39, 58, 115). For instance, TCF1 and Thpok, which are shown to be required for generation of T_CM_ cells, also promotes Tfh cell differentiation during Th1 responses (41, 52, 55, 58). Bcl6, which plays pivotal role in Tfh differentiation, is also required for T_CM_ and Th1-T_RM_ generation (58, 59, 94). Moreover, lack of a unifying approach to faithfully identify Th1/Th2/Th17 and Tfh populations complicates the study of CD4^+^ T_M_ cell differentiation. Th1, Th2 and Th17 cells are generally identified by secretion of IFN-γ, IL-4 and IL-17 respectively; however, Bcl6^+^ follicular helper T (Tfh) cells are also able to produce these hallmark cytokines (34). Besides, the two commonly used approaches, based on expression of Ly6C/PSGL1/FR4 and CXCR6/CXCR5/CCR7, both cannot unambiguously identify the diverse CD4^+^ T cell subsets during effector and memory phases of Th1 responses (32, 37, 39, 45). Expression of CXCR5 and production of IL-4 are also not able to discriminate between Th2 and Tfh cells under parasite infection (101, 102). The developmental relationships between Th/Tfh polarization and memory generation remain incompletely elucidated, largely due to shared mechanistic pathways and phenotypic overlaps between diverse CD4^+^ T cell subsets.

Furthermore, the plasticity of CD4^+^ T cell subsets at population level also increases complexity in studying CD4^+^ T memory generation (116). During Th1 responses, CXCR5^+^ T_M_ cells give rise to secondary Th1 and Tfh effector cells upon rechallenge, indicating heterogeneity or plasticity of them (44). Recent data demonstrate that CXCR5^+^ memory cells contain a T_CM_-like (CCR7^+^ or Ly6C^lo^PSGL1^+^) population, which primarily gives rise to secondary Th1 effectors and generates few Tfh cells (41, 45, 117). However, remaining Tfh memory cells (Ly6C^lo^PSGL1^lo^FR4^+^ or CXCR5^+^CCR7^lo^), which efficiently generate Tfh effector cells, equally give rise to secondary Th1 effectors upon challenge (41, 45). It might be because Tfh-like memory cells indeed possess plasticity, or they still represent a heterogeneous population. In addition, Gata3^+^ Th2 and Bcl6^+^ Tfh cells exhibit great plasticity, as each can give rise to both cell types following helminth challenge (101, 102). On the other hand, a specific CD62L^+^CXCR5^+^Bcl6^+^ Tfh cell population has recently been shown to efficiently generate Tfh effectors during secondary response, indicating lineage commitment of it (86). Besides, Th1-T_EM_ cells (Ly6C^hi^ and/or CXCR5^lo^) almost exclusively generate secondary Th1 effector cells upon rechallenge (38, 42, 44, 45, 49). Some other fate mapping and single-cell studies also indicate that CD4^+^ T_M_ cells exhibit minimal plasticity and might be lineage-committed upon recall (38, 39). A possible explanation is that the plasticity of CD4^+^ T_M_ cells at population level is associated with additional heterogeneity (116). Nonetheless, the plasticity at the population level currently hinders a deeper understanding of CD4^+^ T_M_ cell generation.

## 6 Prospects for identifying potential common precursors of specific CD4^+^ T_M_ cells

As discussed above, CD4^+^ T_M_ cells are suggested to derive from antigen-activated T cell progenitors, and the memory fate is primarily dictated during priming phase of primary response (39, 62). However, the lack of a guiding model significantly hinders further investigations into the mechanisms underlying generation of CD4^+^ T_M_ cells. Identification and characterization of potential common precursors of specific CD4^+^ T_M_ cells under Th1, Th2 and Th17 conditions would be beneficial for further investigating the programs of fate decision between effector and memory CD4^+^ T cells.

Unfortunately, the current theory of CD8^+^ T_M_ cell generation provides only limited guidance for identifying precursors of CD4^+^ T_M_ cells. As a reliable marker for CD8^+^ T_M_ precursors (8, 9), role of CD127 in generation of CD4^+^ T_M_ cells has attracted considerable attentions. Multiple studies demonstrate that CD127 mediated IL-7 signaling is required for maintenance and homeostasis of CD4^+^ T_M_ cells; however, CD127 seems to be downregulated at effector phase and thus cannot identify memory precursors at the peak of Th1, Th2 and Th17 responses (48, 52, 69, 84, 86, 104, 108, 110, 118). Nonetheless, revealing programs that regulate re-expression of CD127 in activated CD4^+^ T cells during contraction phase might be a breakthrough for identifying common memory precursors, as IL-7/CD127signaling has been shown to be essential for survival of activated CD4^+^ T cells during this phase (52, 84, 110, 119). On the other hand, the dual roles of TCF1 in CD4^+^ T_M_ cell differentiation and Tfh polarization make it an unreliable marker for memory precursors during Th1/Tfh response under viral infections (39, 52–57). Besides, RNA-seq data indicates that TCF1 is not involved in formation of T_CM_ precursor-like CD62L^+^ T cells during Th2 response (104). The exact role of TCF1 in memory generation and Th/Tfh lineage polarization still needs to be defined. Collectively, CD127 and TCF1 are not reliable markers for discriminating CD4^+^ T memory precursors from terminally differentiated effectors.

Programs of CD4^+^ T_M_ cell differentiation also exhibit great differences under Th1, Th2 or Th17 conditions (5, 43, 94). Commonly used markers during Th1 responses, such as Ly6C, PSGL1 and CXCR5, seem unable to distinguish between T_CM_ precursors, Th2 effector cells, and Tfh cells under Th2 conditions (101, 102, 104). Besides, whether T_CM_-associated factors during Th1 response, such as TCF1, Thpok and OCA-B, also participate in regulating memory formation under Th2 and Th17 conditions remains ill-defined (32, 58, 104). The mechanisms of T_CM_ and Tfh memory cell differentiation under various types of infections and exposures are far from clear. Moreover, the generation of CD4^+^ T_EM_ and T_RM_ cells are closely related to Th1, Th2, Th17 and even Tfh effector cell differentiation (5, 43, 94). For instance, the Th1 hallmark transcription factor T-bet is indispensable for terminal effector differentiation and the generation of T_EM_ and T_RM_ cells through multiple mechanisms during Th1 responses, whereas it is clearly not involved in effector and memory differentiation under Th2 and Th17 conditions (81, 93, 120). Whether Gata3 and RORγt contributes to T_EM_ and T_RM_ generation during Th2 and Th17 responses remains unclear. It seems that the more we delve into the differences between subsets of CD4^+^ T cells, the harder it becomes to develop a general model of their memory generation.

**Figure 4:**
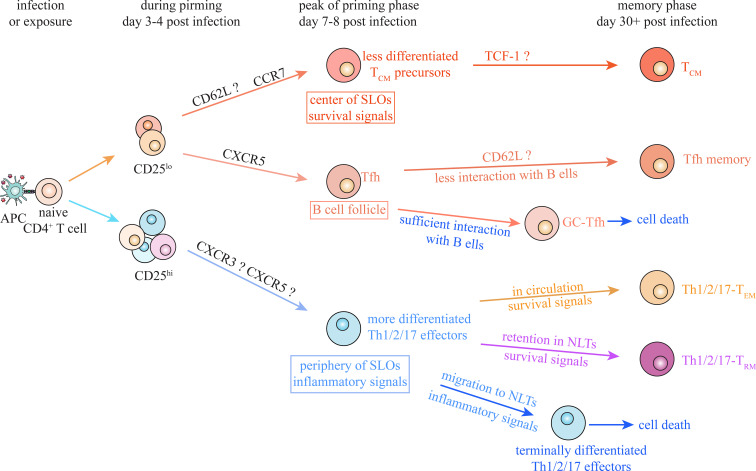
Putative common model of CD4^+^ T_M_ cell generation. Despite the differences between CD4^+^ T cell subsets, there are some common mechanisms underlying memory generation during Th1, Th2 and Th17 responses. IL-2/CD25 signaling contributes to determining the fate choice between Th1/2/17 effector and Tfh/T_CM_ at early stage of priming under multiple conditions. Moreover, migration of activated CD4^+^ T cells toward periphery of secondary lymphoid organs (SLOs) and inflamed non-lymphoid tissues (NLTs), facilitates their encounter with inflammatory signals and thus promotes their effector differentiation. Meanwhile, positioning of activated CD4^+^ T cells into appropriate niches, avoiding excessive stimulation and receiving survival signals, is suggested to be essential for generation of specific CD4^+^ T_M_ subsets under various types of infections and exposures.

Nevertheless, we cannot exclude the possibility that there exists a common mechanism for generation of multiple CD4^+^ T_M_ populations under different conditions. TCR-dependent IL-2/CD25 signaling at early priming stage contributes to Th1/2/17 effector cell differentiation, whereas its absence favors the formation of Tfh and T_CM_ cells during both Th1 and Th2 responses (57, 66, 95, 99, 121). IL-2/CD25 signaling during priming stage is also required for Th1-T_RM_ and Th2-T_RM_ generation through promoting effector differentiation and NLT residency (95, 96, 99). Although it has been argued that IL2/CD25 signaling promotes survival and persistence of CD4^+^ T cells at later stage (69, 70), it indeed contributes to determining the fate choice between Th1/2/17 effector cells and Tfh/T_CM_ cells at the early stage during priming (**Figure 4**). Some other factors, including ACC1 and CD30, have also been shown to participate in regulating the transition of Th1/2/17 effector cells into lineages-committed Th1/2/17 memory cells under multiple conditions (64, 65). It is indicated that activated CD4^+^ T cells undergo some common processes that regulate their transition into memory cells, although decisive factors that dictate the fate decision between effector and memory under diverse conditions have not been revealed.

On the other hand, positioning of activated CD4^+^ T cells, which facilitates their interaction with specific extrinsic signals, plays a critical role in effector and memory differentiation of Th1, Th2, Th17 and Tfh cells (**Figure 4**). Localization in periphery and center of SLOs to interact with inflammatory and survival signals controls effector and T_CM_ differentiation, respectively, during both Th1 and Th2 responses (20, 37, 41, 42, 52, 65, 84, 103). Retention of activated T cells in NLTs to interact with local inflammatory and survival signals is suggested to be indispensable for T_RM_ generation during Th1, Th2 and Th17 responses, though factors regulating their residency in various NLTs remain unclear (93–96, 98, 99, 105, 111, 112, 122). Localization in B cell follicle (and GC area) and insufficient interaction with B cells both are suggested to be required for Tfh memory generation under viral infections and antigen exposures (84, 86). Consistent spatial requirements for differentiation of specific CD4^+^ T_M_ subsets also provide valuable perspectives for identifying common memory precursors under various types of infections and exposures.

## 7 Concluding remarks

CD4^+^ T_M_ cells play critical roles in protective immunity and immunopathology. Revealing underlying mechanisms of their generation and maintenance is crucial for developing therapeutic approaches targeting CD4^+^ T_M_ cells in human diseases. The intriguing MPEC/SLEC model has led to remarkable advances in the understanding of generation of CD8^+^ T_CM_, T_EM_ and T_RM_ cells. However, there is a lack of guiding model for further investigations into generation of CD4^+^ T_M_ cells, due to the diversity and plasticity of multiple CD4^+^ T subsets. Differentiation of specific CD4^+^ T_M_ subsets under Th1, Th2 and Th17 conditions depends on both common and distinct underlying mechanisms. The shared features discussed above might provide a path to a putative common model on generation of CD4^+^T_M_ subsets under all types of infections and exposures. Clearer delineations of Tfh memory, T_CM_, T_EM_ and T_RM_ cell populations at memory stage, as well as Tfh and Th1/2/17 populations at priming stage, are definitely required. Future works are also needed to reveal undergo programs governing memory generation under specific conditions and to clarify the consistency of these programs across different types of infections and exposures. Considering that transcriptional networks and positioning in proper niches to receive extrinsic stimulation both contributes to effector and memory differentiation, integrating spatial transcriptomics and single-cell RNA-seq technology will be an informative approach for further investigations (123). In addition, the advancing MHC-II multimer technology will be a valuable tool for studying endogenous antigen-specific CD4^+^ T cells (124). Identifying potential common and distinct precursors for each CD4^+^ T_M_ cell subset would be highly beneficial for elucidating underlying mechanisms for their generation.

On the other hand, recent findings have suggested that CD8^+^ cytotoxic T (Tc) cells also differentiate into multiple subsets, in a manner similar to CD4^+^ T cells (125). Conventional IFNγ-producing Tc1 cells, IL-4-producing Tc2 cells, and IL-17-producing Tc17 cells, which are polarized by specific cytokine microenvironments, are all capable of differentiating into long-lived T_M_ cells (125–127). However, whether each CD8^+^ T_M_ cell subset derives from distinct or common precursor populations and whether they exhibit plasticity remains largely unclear. Elucidating the mechanisms underlying the generation of multiple CD4^+^ T_M_ cell subsets may provide valuable insights into the differentiation of memory CD8^+^ Tc1, Tc2, and Tc17 cells.
